# Prevalence, knowledge and practices of shisha smoking among youth in Kampala City, Uganda

**DOI:** 10.11604/pamj.2019.32.61.15184

**Published:** 2019-02-05

**Authors:** Christine Aanyu, Daniel Kadobera, Rebecca Racheal Apolot, Angela Nakanwagi Kisakye, Peter Nsubuga, William Bazeyo, John Bosco Ddamulira

**Affiliations:** 1 Makerere University School of Public Health, Kampala, Uganda; 2 Ministry of Health, Uganda; 3 African Field Epidemiology Network, Kampala, Uganda; 4 Global Public Health Solutions, Atlanta, USA

**Keywords:** Shisha smoking, knowledge, practice, youths

## Abstract

**Introduction:**

globally tobacco use kills more than seven million people annually, a figure expected to rise to 8 million deaths every year by 2030. Though perceived as safe, shisha smoking is reported to have the same or worse health effects as cigarette smoking yet, this practice has gained popularity especially among youths globally. We assessed shisha smoking and factors associated with shisha smoking to support public health interventions.

**Methods:**

a cross-sectional study was conducted among 663 systematically selected youths aged between 18-30 years attending bars in two divisions of Kampala city Uganda. Data was analyzed using Stata version 12 and logistic regression model run to establish factors independently associated with shisha smoking.

**Results:**

we found that 458 (86.4%) youths had low knowledge of the health effects of shisha and 193 (36.4%) smoked shisha. Majority of the respondents, 184 (97.4%) smoked flavoured and sweetened tobacco, 69 (36.5%) smoked on a weekly basis, 163 (86.2%) smoked in the company of friends, 162 (85.7%) shared shisha pipes. Factors associated with shisha smoking include smoking cigarettes adjusted odds ratio [aOR]: 5.91, 95% Confidence Interval (CI): 3.86-9.05); positive attitude (aOR: 3.89, 95% CI: 2.50-6.05); urban residence (aOR: 3.98, 95% CI: 1.99-8.00) and older age [25-30 years] (aOR: 2.13, 95% CI: 1.37-3.22).

**Conclusion:**

the prevalence of shisha smoking is high with three in ten youths smoking shisha yet their knowledge about the health effects associated with shisha smoking was low. Shisha smoking ban should be implemented in all bars in Kampala as stated by the newly enacted tobacco law.

## Introduction

Tobacco use is a known health risk associated with cardiovascular diseases, lung cancer, respiratory diseases, and chronic bronchitis [1]. Globally tobacco use kills more than seven million people annually [2]. More than six million of those deaths are as a result of direct tobacco use while 890,000 are the result of exposure to second-hand smoke. Tobacco is used in various forms including cigarettes, cigars, chewable tobacco, bidis, kreteks (also known as clove cigarettes) and “shisha” smoking also known as hookah, water pipe, goza, nargile and hubble-bubble [3]. Shisha smoking is a method of tobacco use which involves passing of smoke through water before inhalation [4-6]. Shisha was invented in India supposedly as a safe method of tobacco use [7]. Although it is true that the water filter system in the shisha pipe may filter out some tobacco-specific carcinogens (i.e., cancer-causing agents) [8], shisha smoke contains many of the same additional toxins as cigarette smoke [9]. Studies have revealed that contrary to the beliefs that shisha smoking is less harmful and less addictive compared to cigarettes, its smoke contains high concentrations of hazardous chemicals and nicotine [1]. Quantities of tobacco by-products found in the blood of shisha smokers are reportedly equivalent to a cigarette smoker who had smoked 10 sticks of cigarette a day [9, 10]. These tobacco hazardous chemicals and by-products expose shisha smokers to a higher risk of diseases such as lung cancer, respiratory diseases, chronic bronchitis, low birth weight, cardiovascular diseases, blindness [8] and nicotine dependence [11-13]. Smokers are also exposed to high levels of carbon monoxide, heavy metals and cancer causing chemicals from the burning of charcoal used for heating shisha. In addition sharing of shisha pipes has been linked to the spread of infectious diseases like hepatitis B, herpes, tuberculosis and flu [14, 15]. Shisha smoking commonly takes place in groups in places of socialisation with bars, cafes and restaurants as the most preferred while other forms of tobacco use tend to occur individually at homes [11, 16, 17]. Peer pressure has been cited as the main reason for initiating shisha smoking [18] because the majority of shisha smokers initiated and practised this habit in the company of friends [16, 17, 19, 20]. Flavored and sweetened tobacco was the most preferred tobacco smoked in shisha and one of the reasons cited for its proliferation. According to Martinasek et al., shisha has gained popularity becoming the tobacco smoking style of the 21 ^st^ century among youths similar to the fashionable cigars in the late 1990s [19]. The social context of its usage, fruity flavours and the misconception of less harm have been put forward to explain the popularity of this method of tobacco use among the youth. Studies conducted in different countries have identified factors associated with shisha smoking like limited knowledge about the health hazards of shisha smoking [12], individual factors including age, sex, education, attitude, residence and peer pressure [21, 22] , among others. Additionally having a parent who smoked and awareness about the complications of shisha smoking was also highlighted [15, 20, 22]. The various studies conducted on shisha smoking mostly looked at university students contrary to the youths found in socialisation places in Uganda. On the other hand, countries, where these studies were conducted, tend to differ in the social and cultural background which is a major determinant of youth behaviours. A paucity of literature exists on shisha smoking among the youth in Uganda. Our study assessed the knowledge, practices and factors associated with shisha smoking among the youth attending bars in Central and Makindye divisions of Kampala city to generate information useful for public health interventions toward shisha smoking.

## Methods

**Study design and site**: we conducted a cross-sectional study in Central and Makindye divisions of Kampala city during April through June 2014. Kampala city is Uganda's national and commercial capital with a total area of 197 km2 and a population of 1,507,080 according to Uganda national population and housing census of 2014 report [23]. Kampala city is divided into five divisions (i.e., Central, Kawempe, Makindye, Nakawa and Rubaga) and is comprised of diverse multinational ethnic groups with over 20 universities and tertiary institutions. It has more than 1,500 bars registered by Kampala Capital City Authority (KCCA) which operate throughout the night as long as customers are available.

**Study population**: our study population included youths aged 18 to 30 years who visited bars in Makindye and Central divisions of Kampala city at the time of the study.

**Sample size determination**: we determined the sample size using Kish Leslie (1965) formula with the following assumptions, a prevalence of 50% since there was no study in a similar setting, and a precision of 5%. A total sample size of 663 participants was obtained.

**Sampling**: we selected Makindye and Central divisions purposively because the two had the highest number of registered bars among the five divisions of Kampala. A list of the registered bars was obtained from KCCA revenue department. There were 264 and 248 registered bars in Central and Makindye divisions respectively giving a total of 512 bars altogether. From the list of registered bars, 128 (Central division-66, and Makindye division-62) were randomly selected. The eligible respondents (i.e., youths 18-30 years) were then sampled systematically by taking every third respondent, and a total of 663 youths were selected for the interviews.

**Inclusion criteria**: we included all youths from the age of 18 to 30 years who attended the sampled bars between 5.00 pm to 11.00 pm on weekdays and 2:00 pm to midnight on weekends in Makindye and Central divisions of Kampala city.

**Exclusion criteria**: we excluded youths who were considered too drunk to participate or those that were unable to communicate.

**Dependent variable**: our dependent variable was shisha smoking. We defined shisha smoking as the consumption of tobacco using shisha. The practices of shisha smoking considered were, the age of initiation, kind of shisha usually smoked, frequency and venue of smoking, duration of a smoking session, companion during initiation, regular use as well as sharing of a shisha pipe.

**Independent variables**: our independent variables included socio-demographic characteristics and environmental factors including age, sex, marital status, level of education, residence, smoking status of the parents and youths, knowledge as well as attitudes of youths towards shisha smoking. We defined knowledge of the health effects as the ability by a respondent to identify diseases associated with shisha smoking based on a list of 10 diseases. Other knowledge aspects included the different forms of tobacco use, the composition of shisha and any health hazards associated with exposure of non-shisha smokers to secondhand smoke from shisha.

**Data collection methods**: we used an interviewer-administered semi-structured questionnaire for data collection. This questionnaire was adapted from a previous study carried out in Pakistan [24]. The questionnaire was written in English and translated into Luganda (the local and common language in Kampala). The research assistants and supervisors were trained on the methodology before data collection to orient them on the objectives and ethics of the study.

**Data management and analysis**: we coded, entered and cleaned data using Epidata 3.02. We then exported to Stata version 12.0 for analysis. We summarised all the variables using descriptive statistics. Knowledge of health effects associated with shisha smoking was measured based on a 10 points scale and later classified. Each correct answer on the list of the diseases was given a value of one, and the incorrect answer was given a value of zero. The variable knowledge was, therefore, the sum of the correctly mentioned diseases from the list. Knowledge was then classified as follows; 6-10 = good knowledge, 5 = satisfactory knowledge, 1-4 = poor knowledge, 0 = no knowledge [25]. Knowledge was further categorised into knowledgeable by combining good and satisfactory knowledge and not knowledgeable by combining poor and no knowledge. A Likert's scale was used to assess attitudes of youths towards shisha smoking. There was a list of thirteen statements regarding shisha smoking and corresponding responses as strongly agree, agree, neither agree nor disagree, disagree and strongly disagree. The responses were scored as strongly agree = 5, agree = 4, neither agree nor disagree = 3, disagree = 2, and strongly disagree = 1. The responses were summed up and a total score obtained for each respondent. The mean score was calculated for each respondent, and those scoring below three were classified as having negative while those scoring above the three were classified as having a positive attitude towards shisha smoking. At the univariate level, we calculated proportions for categorical variables and summarised age using mean and standard deviation. A forward stepwise logistic regression model was used to establish factors independently associated with shisha smoking at multivariable level. We set the level of significance at 5% alpha. All statistical analyses were performed using Stata IC version 12.

**Ethical considerations**: we obtained ethical clearance from Makerere University, School of Public Health Institutional Review Board. Permission was obtained from the Directorate of Public Health and Environment of Kampala Capital City Authority, and the respective town clerks were informed about the study. We obtained written consent from the study participants indicating that the participants had agreed to participate in the study. Participation in this study was voluntary, and there were no incentives given to the participants.

## Results

**Socio-demographic characteristics of the respondents**: out of the 663 participants recruited from 128 bars in the two divisions of Kampala, 530 completed the interviews giving a response rate of 80.0%. The mean age of the respondents was 24.8 years (Standard Deviation (SD) ±3.2), and most 285(53.8%) were of age group 25-30years. Males represented 369 (69.6%) of the respondents. Majority of the youth were of secondary or tertiary education 499 (94.2%), resident in urban areas 458 (86.4%), single 388 (73.2%), and had parents who did not smokers 363 (68.5%) (Table 1).

**Table 1:** socio-demographic characteristics of the youths in Kampala by gender, in April-June 2014

Variable	Female	Male	Total
	n=161(%)	n=369(%)	N=530
**Age**			
18-24	89 (36.3)	156 (63.7)	245(46.2)
25-30	72 (25.3)	213 (74.7)	285(53.8)
**Marital Status**			
Single	122 (31.4)	266 (68.6)	388(73.2)
Married	23 (20.0)	92 (80.0)	115(21.7)
Separated/divorced/widowed	16 (59.2)	11 (40.7)	27(5.1)
**Level of education**			
None	3 (60.0)	2 (40.0)	5(0.9)
Primary	9 (34.6)	17 (65.4)	26(4.9)
Secondary	82 (29.5)	196 (70.5)	278(52.5)
Tertiary	67 (30.3)	154 (69.7)	221(41.7)
**Residence**			
Rural	22 (30.6)	50 (69.4)	72(13.6)
Urban	139 (30.3)	319 (69.7)	458(86.4)
**Parents smoking status**			
Yes	40 (24.0)	127 (76.0)	167(31.5)
No	121 (33.3)	242 (66.7)	363(68.5)

**Knowledge on shisha smoking among youths in Kampala**: two thirds, 359 (67.7%) of the participants mentioned between one to four diseases associated with shisha smoking while only 37 (7.0%) of the respondents cited six diseases and above. On further classification of knowledge into low and high, the majority of youths, 458 (86.4%) had low knowledge of the health effects associated with shisha smoking. Among other knowledge components, 301 (56.8%) of the respondents reported that tobacco was one of the components in shisha smoking. A total of 483 (86.7%) respondents reported that shisha smoking had health hazards and 358 (73.0%) of the respondents stated that exposure of non-smokers to second-hand smoke had health hazards (Table 2).

**Table 2:** knowledge of different aspects of shisha smoking among youths in Kampala, in April-June 2014

Knowledge aspects	Response	Female n=161 (%)	Male n=369 (%)	Total N=530
**Shisha smoking has health hazards**	Yes	121 (75.2)	312 (84.6)	433(86.7)
	No	40 (24.8)	57 (15.4)	97(18.3)
**Mentioned Shisha as a form of tobacco use**	Yes	86 (53.4)	272 (73.7)	358(67.5)
	No	75 (46.6)	97 (26.3)	172(32.5)
**Know that Shisha has tobacco as one of its components**	Yes	84 (52.2)	217 (58.8)	301(56.8)
	No	77 (47.8)	152 (41.2)	229(43.2)
**Mentioned health hazards associated with exposure of non-shisha smokers to Second-hand smoke**	Yes	110 (68.3)	277 (75.1)	387(73.0)
	No	51 (31.7)	92 (24.9)	143(27.0)
**Level of Knowledge**	Good	11(29.7)	26(70.3)	37(7.0)
	Satisfactory	6(17.1)	29(82.9)	35(6.6)
	Low	103(28.7)	256(71.3)	359(67.7)
	None	41(41.4)	58(58.6)	99(18.7)

**Practices towards shisha smoking among youths**: about half 268 (50.6) of the respondents smoked any form of tobacco while 193 (36.4%) smoked shisha. The 193 youth who smoked shisha were assessed for the different practices of shisha smoking. Nearly all shisha smokers 184 (97.4%) smoked flavoured and sweetened shisha. Majority 163 (86.2%) of the respondents smoked shisha in the company of friends and 162 (85.7%) of the respondents shared a shisha pipe. Among the respondents who shared a shisha pipe, approximately three quarters 122 (73.1%) were male. The mean age for initiation of shisha smoking was 22.8 years (SD±4.5 years) (Table 3).

**Table 3:** the distribution of shisha smokers according to various characteristics among youths in Kampala, in April-June 2014

Characteristic		Female n=45 (%)	Male n=144 (%)	Total N=189(%)
**Kind of Shisha smoked**	Sweetened and flavoured	45(24.5)	139(75.5)	184(97.4)
	Non sweetened and flavoured	0(0.0)	5(100)	5(2.6)
**Companion at initiation**	Alone	3(21.4)	11(78.6)	14(7.4)
	Friend(s)	38(23.2)	126(76.8)	164(86.8)
	Family	4(36.4)	7(63.6)	11(5.8)
**Frequency of smoking**	Daily	11(18.6)	48(81.4)	59(31.2)
	Weekly	18(26.1)	51(73.9)	69(36.5)
	Monthly/Occasionally	16(26.2)	45(73.8)	61(32.3)
**Smoking venue**	Bars/Restaurants/cafes	42(24.4)	130(75.6)	172(91.0)
	Friend's residence	3(18.8)	13(81.2)	16(8.5)
	Own residence	0(0.0)	1(100)	1(0.5)
**Smoking duration**	<30 minutes	21(20.6)	81(79.4)	102(54.0)
	31-60 minutes	21(28.4)	53(71.6)	74(39.2)
	>60 minutes	3(23.1)	10(76.9)	13(6.9)
**Sharing shisha pipe**	Yes	40(24.7)	122(73.1)	162(85.7)
	No	5(18.5)	22(81.5)	27(14.3)
**Current shisha smoking partner**	Alone	4(19.0)	17(81.0)	21(11.1)
	Friends	38(23.3)	125(76.7)	163(86.2)
	Family	3(60.0)	2(40.0)	5(2.6)

**Factors associated with shisha smoking among youths in Kampala**: bivariable analysis showed an association between shisha smoking with gender, parents' smoking status and marital status but these associations were lost after controlling for other factors. The factors which remained significant at multivariable analysis were cigarette smoking (adjusted odds ratio ((aOR): 5.91, 95% confidence interval (CI): 3.86-9.05); positive attitude (aOR: 3.89, 95% CI: 2.50-6.05); place of residence (aOR: 3.98, 95% CI: 1.99-8.00) and older age (25-30 years) (aOR: 2.13, 95% CI: 1.37-3.22) (Table 4).

**Table 4:** showing factors associated with shisha smoking among youths in Kampala, in April-June 2014

Independent Variable	Odds Ratio (95%CI)			
	Crude	P-value	Adjusted	P-value
**Cigarette smoking status**				
No	1.00		1.00	
Yes	6.11(4.13-9.04)	<0.001	5.91(3.86-9.05)	<0.001
**Attitudes**				
Negative	1.00		1.00	
Positive	4.13(2.80-6.10)	<0.001	3.89(2.50-6.05)	<0.001
**Place of residence**				
Rural	1.00		1.00	
Urban	2.21(1.23-3.98)	<0.001	3.98(1.99-8.00)	<0.001
**Age**				
18-24	1.00		1.00	
25-30	1.91(1.33-2.75)	<0.001	2.13(1.37-3.22)	0.001
**Sex**				
Female	1.00		1.00	
Male	1.71(1.14-2.56)	0.009	1.58(0.98-2.53)	0.061

## Discussion

Findings from our study revealed that there was a high prevalence of shisha smoking among youths attending bars in Kampala city with three in every ten youths smoking shisha. A greater proportion of youths, who attended bars were male, had low knowledge of the health effects associated with shisha smoking and smoked in the company of friends. Among the youths who smoked shisha, majority smoked in the bars and shared shisha pipes. Our study also reported cigarette smoking, positive attitudes, residence and older age (25-30 years old) as factors significantly associated with shisha smoking. The high prevalence of shisha smoking among youths attending bars in Kampala city might be an indication that majority of persons visiting bars are active smokers or exposed to second-hand smoke. It might also point to the fact that the ban on shisha smoking is partially or not fully implemented by the hospitality sector in Uganda. Our findings are similar to findings from studies in Malaysia and Turkey which found a high prevalence of shisha smoking among university students, medical and non-medical [20, 21]. However, our findings were different from the findings of a study conducted in South Africa, among health science students which reported a low prevalence of shisha smoking [25]. We found that youths had insufficient knowledge regarding the harmful effects associated with shisha smoking. This lack of knowledge about the health effects associated with shisha smoking and the diverse population found in Kampala could have led to the proliferation of this type of tobacco use. A study in the United States revealed that exposure of tobacco naïve non-smokers to second-hand smoke exposed them to nicotine doses in sweet smelling and easily inhaled smoke [16]. Similar findings were reported in other studies conducted in South Africa and Malaysia where respondents reported having insufficient knowledge on the hazardous effects of shisha smoking [20, 26]. Public health officials and policymakers should create awareness programs and messages tailored towards youths attending bars. Our study further found out that shisha smoking was practiced in bars in the company of friends sharing shisha pipes mainly on a weekly basis. These findings show that the youths practice social smoking in social settings and the importance of peers in influencing risky behaviors. Similar findings were reported in Pakistan and San Francisco Bay Area, which cited bars, cafes, restaurants and lounges as the preferred places for smoking shisha [17, 27]. However, respondents from a university in South Africa reported that they smoked at the University campus [26]. Sharing of shisha pipes not only expose shisha smokers to infectious agents which cause the spread of communicable diseases but also increases the risk of getting diseases like tuberculosis, flu and herpes [19, 28]. Targeted awareness messages on the dangers of sharing of a shisha pipe need to be created to educate the youths.

Preference of the sweetened and flavoured shisha tobacco was also one of the characteristics exhibited by shisha smokers in our study. The pleasant taste and aroma of shisha tobacco has been reported as one of the reasons leading to the introduction of none smokers to this practice [29]. Previous findings from the United States and Jordan reported that the majority of the participants smoked sweetened and flavoured tobacco [15, 16]. Our study also found out that a cigarette smoker had higher odds of smoking shisha compared to a non-cigarette smoker. This finding implies that being involved in one risky behaviour increases the risk of being involved in other risky behaviours. Our finding is similar with other study findings conducted on shisha smoking by cigarette smokers in United States, Jordan and British University [30-32]. We also found that positive attitude towards smoking was another factor associated with shisha smoking. This could be explained by the fact that having a positive perception towards a particular practice would increase the likelihood of that practice being taken up. Similar findings were reported from a study conducted among college students in South Florida indicated that positive attitudes towards shisha smoking were strongly associated with increased odds of being a current user and of intention to use shisha tobacco in the future [33]. Urban residence was another factor that was associated with shisha smoking. The high prevalence of shisha smoking among urban youth could imply that the trends in urban setting easily shape the youths' culture due to exposure and easy access to information through the media in urban areas both written and electronic. Also, the multinationals that practice this method of smoking are found in urban areas; this could have led the youths in urban areas to follow the trend. A study in Madagascar [34] reported similar findings, but in Vietnam [35], the findings were different where rural residence was associated with shisha smoking. In Turkey, however, the residence was not significantly associated with shisha smoking [21]. Our study had some limitations. Being cross-sectional in nature, could not make causal assumptions. Recall bias was another limitation due to self-reports of the survey responses. This study could have also overestimated the prevalence of shisha smoking due to the homogeneous nature of the study participants. However, these study findings can be generalizable to youths attending bars in Kampala capital city and provides information on this method of tobacco use that could assist policymakers and public health professionals to make targeted interventions.

## Conclusion

Shisha smoking is high among youths attending bars in the two divisions of Kampala yet knowledge of the health effects associated with shisha smoking is limited. A greater proportion of youths, who attended bars were male and smoked in the company of friends. Majority of the youths, who smoked, did so in the bars and shared shisha pipes. Cigarette smoking, positive attitudes, residence and older age (25-30 years old) were found to be significantly associated with shisha smoking. We recommend full implementation of the tobacco control law and sensitisation of the hospitality sectors that hosts the shisha smoking.

### What is known about this topic

- Shisha smoking just like any other tobacco product is a preventable cause of death, killing more than seven million people annually, a figure expected to rise to 8 million deaths every year by 2030;
- Shisha smoking commonly takes place in places of socialization like bars, cafes and restaurants;
- Peer pressure and flavored and sweetened tobacco have been cited as the reasons for its proliferation.

### What this study adds

- Shisha smoking just like any other tobacco product is a preventable cause of death, killing more than seven million people annually, a figure expected to rise to 8 million deaths every year by 2030;
- Shisha smoking commonly takes place in places of socialization like bars, cafes and restaurants;
- Peer pressure and flavored and sweetened tobacco have been cited as the reasons for its proliferation.

## Competing interests

The authors declare no competing interests.
